# Marine Compound Xyloketal B Reduces Neonatal Hypoxic-Ischemic Brain Injury

**DOI:** 10.3390/md13010029

**Published:** 2014-12-24

**Authors:** Ai-Jiao Xiao, Wenliang Chen, Baofeng Xu, Rui Liu, Ekaterina Turlova, Andrew Barszczyk, Christopher Lf Sun, Ling Liu, Marielle Deurloo, Guan-Lei Wang, Zhong-Ping Feng, Hong-Shuo Sun

**Affiliations:** 1 Department of Physiology, Faculty of Medicine, University of Toronto, Toronto, ON M5S 1A8, Canada; E-Mails: xaj527@163.com (A.-J.X.); wenliang.chen@utoronto.ca (W.C.); xubf@jlu.edu.cn day (B.X.); liur@jlu.edu.cn (R.L.); e.turlova@mail.utoronto.ca (E.T.); andrew.barszczyk@utoronto.ca (A.B); christopher.sun@mail.utoronto.ca (C.L.S.); liuling1978@163.com (L.L.); marielle.deurloo@gmail.com (M.D.); 2 Department of Surgery, Faculty of Medicine, University of Toronto, Toronto, ON M5S 1A8, Canada; 3 Department of Pharmacology, Faculty of Medicine, University of Toronto, Toronto, ON M5S 1A8, Canada; 4 Department of Pharmacology, Zhongshan School of Medicine, Sun Yat-Sen University, Guangzhou 510080, China; E-Mail: wangglei@mail.sysu.edu.cn; 5 Institute of Medical Science, Faculty of Medicine, University of Toronto, Toronto, ON M5S 1A8, Canada

**Keywords:** hypoxic-ischemic injury, infarct volume, neuroprotection, oxygen glucose deprivation, primary neuronal cell culture, neonatal stroke, behavioral tests, marine drug

## Abstract

Neonatal hypoxic-ischemic encephalopathy causes neurodegeneration and brain injury, leading to sensorimotor dysfunction. Xyloketal B is a novel marine compound isolated from a mangrove fungus Xylaria species (no. 2508) with unique antioxidant effects. In this study, we investigated the effects and mechanism of xyloketal B on oxygen-glucose deprivation-induced neuronal cell death in mouse primary cortical culture and on hypoxic-ischemic brain injury in neonatal mice *in vivo*. We found that xyloketal B reduced anoxia-induced neuronal cell death *in vitro*, as well as infarct volume in neonatal hypoxic-ischemic brain injury model *in vivo*. Furthermore, xyloketal B improved functional behavioral recovery of the animals following hypoxic-ischemic insult. In addition, xyloketal B significantly decreased calcium entry, reduced the number of TUNEL-positive cells, reduced the levels of cleaved caspase-3 and Bax proteins, and increased the level of Bcl-2 protein after the hypoxic-ischemic injury. Our findings indicate that xyloketal B is effective in models of hypoxia-ischemia and thus has potential as a treatment for hypoxic-ischemic brain injury.

## 1. Introduction

Neonatal hypoxic-ischemic encephalopathy (HIE) is a childhood brain disorder that affects up to 2 per 1000 neonates [1,2] and remains a significant health issue for newborns and children, with poor prognosis, high mortality and significant disability. Perinatal and neonatal hypoxic-ischemic brain injury leads to HIE and its related brain disorders such as cerebral palsy, epilepsy, learning disability, memory retardation, and other neurological abnormalities [3,4]. The disabilities in afflicted survivors of infant hypoxic-ischemic brain injury result in a significant social and economic burden worldwide. There is no effective treatment for the neonatal hypoxic-ischemic brain injury. Therefore, it is important to identify new drug targets for prevention and/or reduction of brain injury associated with hypoxic-ischemic insult in neonatal stages.

**Scheme 1:**
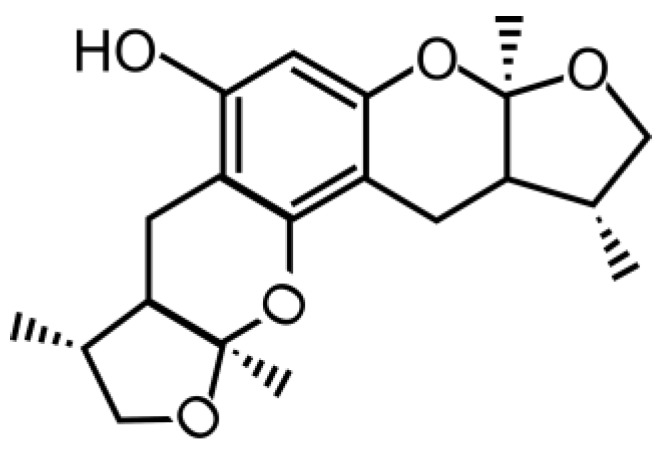
Chemical structure of xyloketal B.

Xyloketal B is a novel marine compound isolated from mangrove fungus *Xylaria* sp. (strain no. 2508) obtained from the South China Sea [5,6,7,8]. It has unique structural features (Scheme 1) [5], exhibits antioxidative effects [6,9,10], and protects cells against injury under different conditions *in vitro* [9,11,12,13]. It enhances nitric oxide production against ox-low density lipoprotein (LDL)-oxidative injury in human umbilical vein endothelial cells [6,9,10]. It also increases cell survival following 1-methyl-4-phenylpyridinium (MPP+)-induced neurotoxicity in PC-12 cells [11], *Caenorhabditis elegans* [11,12] and mouse dopaminergic neurons [12]. More importantly, xyloketal B reduces oxygen-glucose deprivation (OGD)-induced cell injury in PC12 cells *in vitro* [13], leading to the possibility that xyloketal B may have neuroprotective effects against hypoxic-ischemic neuronal injury *in vitro* and/or *in vivo*. Xyloketal B has not yet been tested previously for its neuroprotective effects and therapeutic potential for stroke using both *in vitro* OGD with mouse primary neuronal cell culture and *in vivo* animal stroke models. In order to advance this marine drug xyloketal B in potential drug development for stroke treatment, we have, in first time, evaluated the drug both *in vitro* and *in vivo* using primary neurons in OGD and mouse neonatal hypoxic-ischemic brain injury model, respectively.

In this study, we used hypoxic-ischemic brain injury *in vivo* and anoxia-induced neuronal cell death (OGD) *in vitro* to investigate the potential neuroprotective effects of xyloketal B on mouse neurons. Oxidative stress is associated with caspase activation leading to apoptosis. Apoptosis is involved in both HIE and ischemic brain injury in neonatal models [14,15]. Thus, we also investigated whether xyloketal B inhibits a pro-apoptotic signaling pathway in hypoxic-ischemic neuronal injury in neonates.

## 2. Results and Discussion

### 2.1. Xyloketal B Reduces OGD-Induced Cell Death in Primary Cortical Culture

**Figure 1:**
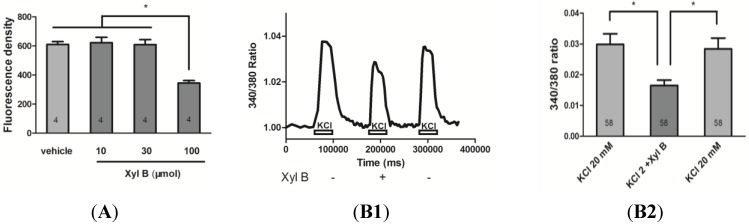
Neuroprotective effects of xyloketal B in cortical neuronal cell culture *in vitro*. (**A**) Xyloketal B reduced OGD-induced primary cortical cell death. Cells were grown in 96-well plates followed by treatment with the indicated concentrations of xyloketal B or vehicle (0.1% DMSO) for 30 min and then subjected to OGD for 90 min. Cell death was determined using propidium iodide (PI, 1 μg/mL) staining. Fluorescence density of PI was detected with a microplate reader. Xyloketal B (100 μM) significantly decreased fluorescence density as compared to the other groups. Data are presented as mean ± S.E.M. * indicates *p* < 0.05, *n* = 4. (**B**) Fura-2 ratiometric calcium imaging showing that xyloketal B reduced KCl-induced calcium entry in primary cortical neurons. (**B1**) Representative and (**B2**) summary of fura-2 calcium signals in cortical neurons (day *in vitro* 6, DIV 6) were incubated with vehicle or xyloketal B (100 μM), followed by 20 mM KCl-induced calcium influx detection using fura-2 calcium indicator. Incubation of xyloketal B (100 μM) reduced calcium influx caused by membrane depolarization by 34% ± 9% of the vehicle treatment. *: *p* < 0.05, *n* = 58.

We first investigated whether xyloketal B reduced OGD-induced neuronal death in primary cortical cell culture. Various concentrations of xyloketal B were added to primary mouse cortical culture at 7 days *in vitro* (DIV) 30 min before OGD conditions. Cells were then stained with propidium iodide (PI) and the fluorescence density of PI staining was measured to indicate the level of cell death. As shown in Figure 1A, the fluorescence density (arbitrary unit) of cells pretreated with 100 μM xyloketal B was significantly decreased (344.50 ± 17.12, *n* = 4, *p* < 0.05) in comparison to cells pretreated with vehicle (610.25 ± 19.54, *n* = 4), or lower xyloketal B concentrations (10 μM: 622 ± 37.77; 30 μM: 608.75 ± 34.96; *n* = 4). This result indicates that pre-treatment with 100 μM xyloketal B reduced OGD-induced cell death *in vitro*.

### 2.2. Xyloketal B Reduces Calcium Entry in Primary Cortical Neurons

Cell death induced by OGD is largely associated with calcium overload [16,17]. To test whether xyloketal B exerts its effect by affecting calcium entry, we measured calcium signals in cultured cortical neurons using ratiometric fura-2 calcium-sensitive dye. Specifically, fura-2 calcium signals were acquired during membrane depolarization by 20 mM KCl with or without 100 μM xyloketal B (Figure 1B1). As shown in Figure 1B2, xyloketal B significantly reduced calcium influx compared to the control by 34% ± 9% (*p* < 0.05). The inhibitory effect of xyloketal B was reversible, as calcium signal returned to the control level after xyloketal B was washed out. Our results suggest that xyloketal B reduces OGD-induced cell death at least in part due to a decrease in calcium entry.

### 2.3. Xyloketal B Reduces Hypoxic-Ischemic Brain Injury and Improved Behavioral Performance

**Figure 2:**
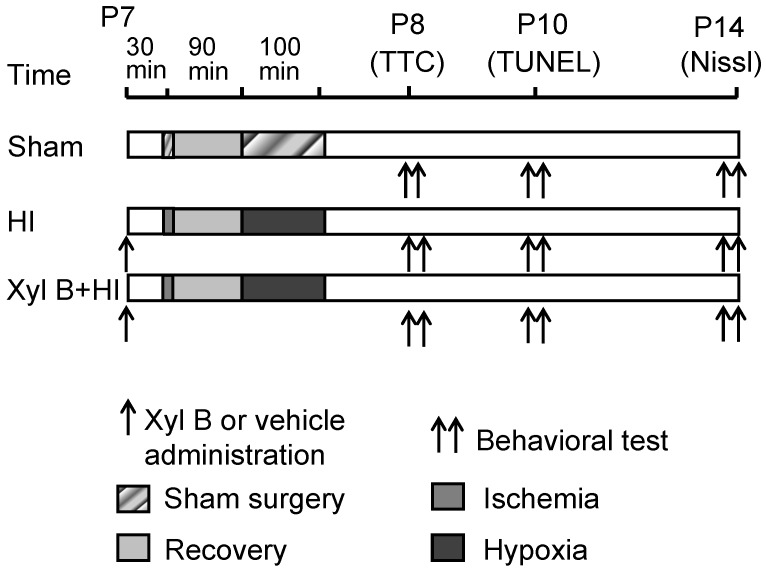
Experimental design and timelines. Sham: Control animals underwent general anesthesia and sham surgical procedure without ischemia and hypoxia. HI: hypoxic-ischemic animals underwent ischemia induced by right common carotid artery occlusion followed by 100 min of hypoxia (8% oxygen balanced with 92% nitrogen). Xyl B + HI: hypoxic-ischemic animals with xyloketal B pretreatment 30 min prior to the onset of HI. Arrows: indicate the time of Xyl B or vehicle administration. Double arrows indicate the time that behavioral tests were conducted at 1 (P8), 3 (P10) and 7 (P14) days after the HI. P7 = postnatal 7 days; TTC stain was performed 24 h after HI at P8; TUNEL stain was performed 3 days after HI at P10; whole brain imaging and Nissl stain were performed 7 days after the HI at P14.

To investigate whether xyloketal B is neuroprotective *in vivo*, a hypoxic-ischemic (HI) brain injury model was employed, and the protocol was described in Figure 2.

#### 2.3.1. Infarct Volume

**Figure 3:**
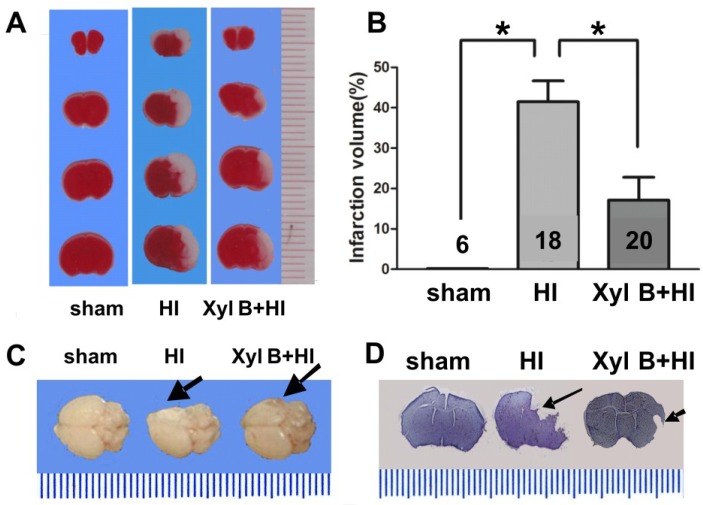
Effects of xyloketal B on infarct volume from neonatal HI brain injury. (**A**) Representative brain coronal sections stained with 2,3,5-triphenyltetrazolium chloride (TTC). White staining indicates damage areas. Xyloketal B pretreatment decreased brain infarction size of HI mice in comparison to that of the vehicle-treated group. Brain infarct volume was detected 24 h after HI injury; (**B**) Analysis of brain infarct volumes of the sham, HI-treated with vehicle or HI-treated with xyloketal B. The percentage of ischemic lesion area was calculated as follows: Corrected infarct volume(%) = [contralateral hemisphere volume-(ipsilateral hemisphere volume-infarct volume)]/contralateral hemisphere volume × 100%. Xyloketal B pretreatment decreased brain infarct volumes in comparison with that of the vehicle-treated group. *: *p* < 0.05, *n* = 6–20; (**C**) Representative whole brains in 7 days after hypoxic-ischemic (HI) insult. Arrows indicate injury sites. Xyloketal B pretreatment reduced brain damage in long term (showing complete brain) in comparison to vehicle-treated HI group (showing the missing brain tissue); (**D**) Representative brain coronal sections stained with cresyl violet in 7 days after HI insult. Arrows indicate injury sites. Xyloketal B pretreatment reduced brain damage in long term (showing coronal section) in comparison the vehicle-treated HI group (missing tissue).

Infarct volumes were measured from the sham or HI groups (vehicle 2% DMSO, i.p.) with or without xyloketal B (5 mg/kg body weight in 0.1 mL, i.p) pre-treatment. Brain slices obtained from all groups were stained with TTC (2,3,5-triphenyltetrazolium chloride) 24 h after HI (Figure 3A). Xyloketal B significantly reduced infarct volume (Xyl B + HI group: 17.10% ± 5.68%. *p* < 0.05) when compared to the vehicle-treated HI group (41.48% ± 5.18%) (Figure 3B). Dramatic morphological changes of whole brains were also observed 7 days post-HI (Figure 3C). While the sham group had normal gross anatomy of the brain, the HI group had damaged ipsilateral brain hemispheres showing missing or deformed regions, and the Xyl B + HI group had less damage, with near-normal morphology. Brain slices stained with Nissl stain (cresyl violet) 7 days post-HI also showed less damage in the Xyl B + HI group compared to that of HI group (Figure 3D). These results demonstrate that xyloketal B pre-treatment significantly reduced subsequent brain damage following HI insult in the neonatal model *in vivo*.

#### 2.3.2. Body Weight

**Figure 4:**
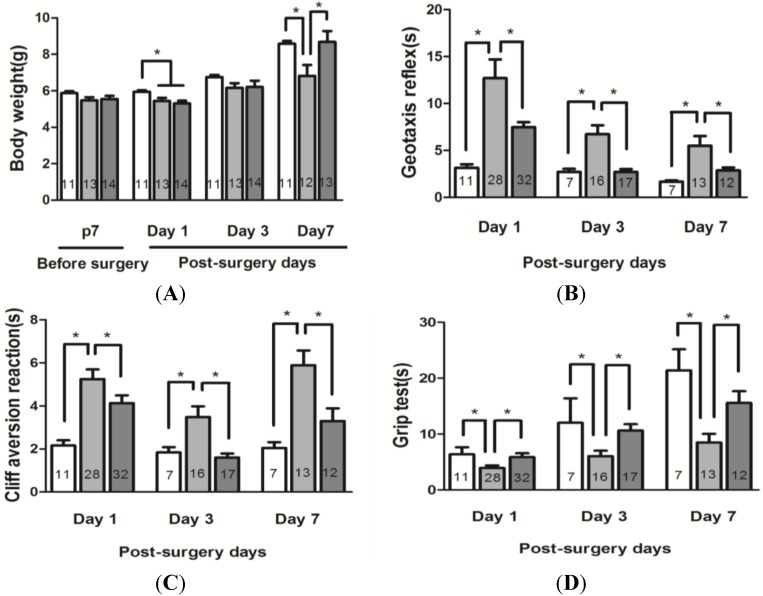
Effects of xyloketal B on growth and functional recovery in neonatal HI brain injury. (**A**) Body weights of the pups in 7 days after HI in comparison to the vehicle-treated HI group; (**B**) Geotaxis reflex. Summary of the data. Xyloketal B improved geotaxis reflex in 1, 3 and 7 days after HI in comparison to the vehicle-treated HI group; (**C**) Cliff aversion reaction. Summary of the data. Xyloketal B improved cliff aversion reaction in 1, 3 and 7 days after HI in comparison with the vehicle-treated HI group; (**D**) Grip test. Summary of the data. Xyloketal B improved grip test in 1, 3 and 7 days after HI in comparison with the vehicle-treated HI group. *: *p* < 0.05, *n* = 7–32. sham: Sham-surgery group; HI: Hypoxic-ischemic injury group; Xyl B + HI: hypoxic-ischemic injury plus pretreatment with xyloketal B group.

Body weight is frequently used as an indicator of the general health of mouse pups. Pups were randomly assigned to different experimental groups, and body weight of the animals was compared on postnatal day 7 prior to the onset of HI, then 1, 3, and 7 days after HI between sham, vehicle-treated HI and xyloketal B-treated HI groups (Figure 4A). Mean body weight was significantly reduced in animals on day 1 following HI protocols, as compared to the sham controls, while no significant difference was observed on postnatal day 7 prior to the onset of HI 5.87 ± 0.11 g (sham group), 5.47 ± 0.18 g (vehicle treated HI group) and 5.54 ± 0.17 g (Xyl B + HI group). Seven days after HI, mice in sham (8.58 ± 0.15 g) and xyloketal B-treated groups (8.68 ± 0.60 g) gained significantly more weight than vehicle-treated HI mice (6.83 ± 0.59 g. *p* < 0.05). These results indicated that xyloketal B promoted general health recovery after the HI procedure.

#### 2.3.3. Neurobehavioral Tests

We next determined whether xyloketal B improves neurological functional recovery by assessing a battery of characteristics, including geotaxis reflex, cliff avoidance reaction, and grip ability. All the tests were carried out in pups in sham, HI and HI + Xyl B-treated groups at various time points before and following HI (Figure 2).

Geotaxis Reflex. Mouse pups in the vehicle-treated HI group (1 day: 12.69 ± 2.01 s; 3 days: 6.72 ± 0.96 s; 7 days: 5.49 ± 1.02 s) exhibited a significantly longer latency to complete the reflex than the sham group (1 day: 3.15 ± 0.38 s; 3 days: 2.70 ± 0.31 s; 7 days: 1.67 ± 0.13 s). Mice in xyloketal B-treated group (1 day: 7.47 ± 0.53 s; 3 days: 2.70 ± 0.32 s; 7 days: 2.86 ± 0.31 s) exhibited a significant reduction in the latency at 1 day, 3 days and 7 days in compared to the vehicle-treated group (Figure 4B, *p* < 0.05). The results indicate that HI causes an impairment in the geotaxis reflex and xyloketal B improves the performance of mice over 7 days post-HI.

Cliff Avoidance Reaction. Mice in the vehicle-treated HI group (1 day: 5.25 ± 0.44 s; 3 days: 3.49 ± 0.50 s; 7 days: 5.89 ± 0.68 s.) required significantly more time to respond to the cliff than mice in the sham group at all time points tested (1 day: 2.16 ± 0.23 s; 3 days: 1.83 ± 0.25 s; 7 days: 2.05 ± 0.27 s). Mice treated with xyloketal B (1 day: 4.09 ± 0.36 s; 3 days: 1.61 ± 0.19 s; 7 days: 3.30 ± 0.59 s) exhibited a significant reduction in latency at 1 day, 3 days and 7 days compared to the vehicle-treated HI group (Figure 4C, *p* < 0.05). These results indicate that xyloketal B improves cliff avoidance after HI.

Grip Test. Mice in the vehicle-treated HI group showed reduced grip ability at all three time points after HI (1 day: 3.94 ± 0.45 s; 3 days: 6.03 ± 0.99 s; 7 days: 8.47 ± 1.56 s), compared to the sham control group (1 day: 6.37 ± 1.25 s; 3 days: 11.83 ± 3.68 s; 7 days: 21.38 ± 3.78 s) (Figure 4D). Xyloketal B treatment improved the grip ability of mice (1 day: 5.87 ± 0.69 s; 3 days: 10.61 ± 1.17 s; 7 days: 15.55 ± 2.10 s) in comparison with the vehicle-treated HI group, which exhibited significantly shorter grip time. (Figure 4D, *p* < 0.05). Our results indicate that pre-treatment with xyloketal B improved grip test performance for all three time points.

### 2.4. Effects of Xyloketal B on Apoptosis

#### 2.4.1. Xyloketal B Inhibits HI-induced Apoptosis

**Figure 5:**
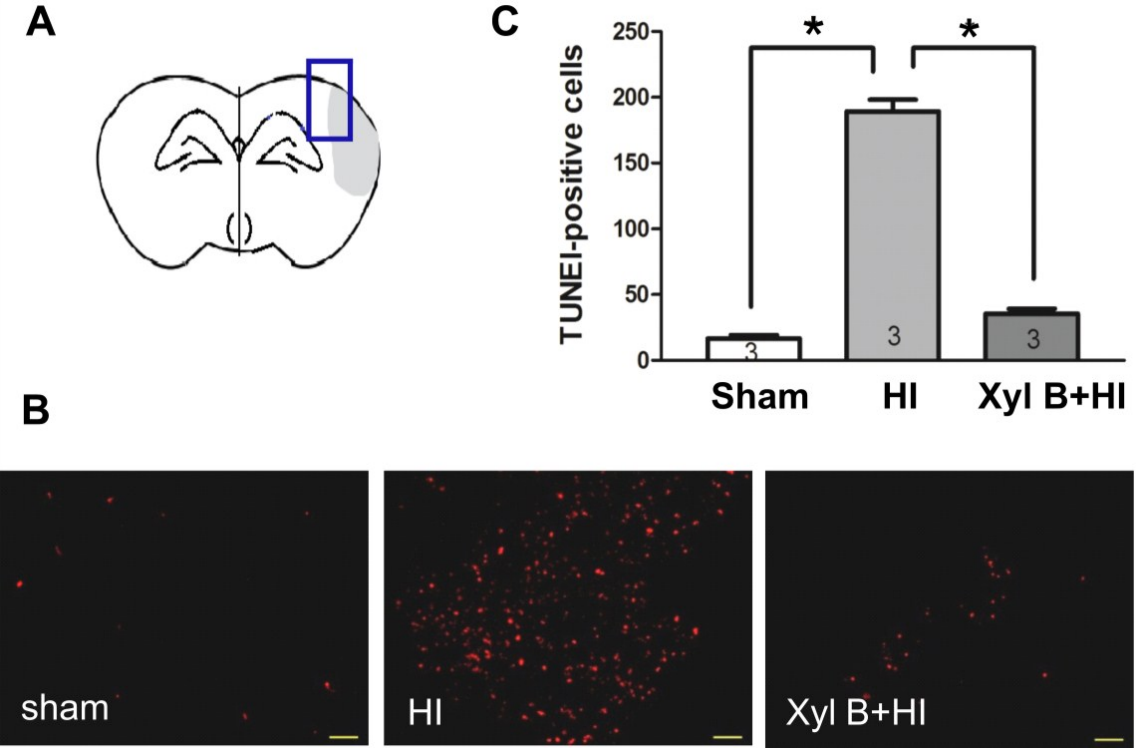
Effects of xyloketal B on apoptosis in neonatal HI brain injury. (**A**) Mouse brain coronal section model image. Blue box: TUNEL staining area; (**B**) Apoptotic cells detected by the TUNEL assay. Images showed representative TUNEL-positive cells (red) of sham, HI only and xyloketal B treated brains. Scale bar corresponds to 50 µm; (**C**) Quantitative analysis of TUNEL-positive cells of sham, HI only and xyloketal B treated brains. The TUNEL positive cells were counted per ×10 field in the penumbra area. Xyloketal B pretreatment reduced the number of TUNEL-positive cells in comparison to the HI only group in 3 days after HI. *: *p* < 0.05, *n* = 3. Sham: Sham-surgery group; HI: Hypoxic-ischemic injury group; Xyl B + HI: hypoxic-ischemic injury plus pretreatment with xyloketal B group.

We next investigated the potential mechanisms underlying the neuroprotective effects of xyloketal B. We first asked whether xyloketal B reduced HI-induced apoptosis. Fragmentation of nuclear DNA in the penumbral area in the ipsilateral brain (Figure 5A) was measured by TUNEL (terminal deoxynucleotidyl transferase dUTP nick-end labeling) staining 3 days after HI insult (Figure 5B). A count of TUNEL-positive cells from the ipsilateral hemisphere showed that the xyloketal B pre-treatment significantly decreased the number of TUNEL-positive cells (Xyl B + HI group: 35.50 ± 3.80 TUNEL positive cells per × 10 field. *p* < 0.05), compared to the vehicle-treated HI group (189.17 ± 8.99 TUNEL positive cells per × 10 field.), while the sham group had 16.67 ± 2.59 TUNEL positive cells per × 10 field (Figure 5C). This result indicates that xyloketal B pre-treatment inhibited HI-induced apoptosis.

#### 2.4.2. Xyloketal B Inhibits the Expression of Apoptosis-Associated Proteins

**Figure 6:**
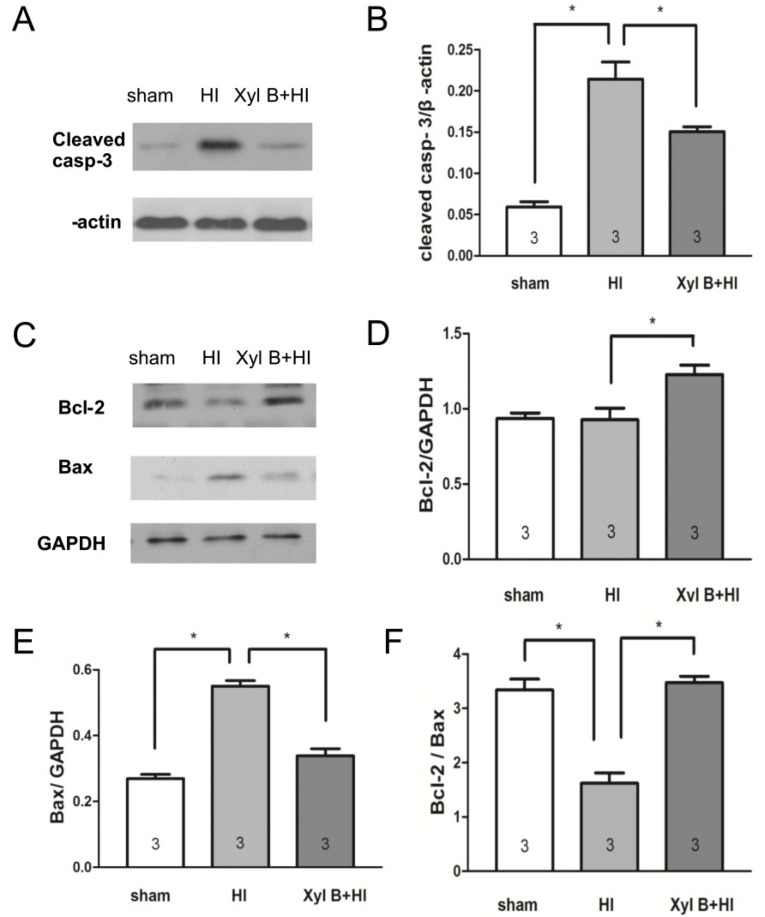
Western blot analysis showing effects of xyloketal B on the expression of apoptosis-related proteins in neonatal HI brain injury. Proteins were extracted from ipsilateral brain of mice. (**A**). Representative western blot showing the level of cleaved caspase-3 protein 1 day after HI insult; (**B**) Densitometric analysis of cleaved caspase-3 bands of sham, vehicle-treated HI- and xyloketal B-treated HI brains. Xyloketal B pretreatment decreased the expression of cleaved caspase-3 (cleaved casp-3) proteins 24 h after HI in comparison to the vehicle-treated HI group; (**C**) Representative western blot showing the level of Bcl-2 and Bax protein 1 day after HI insult; (**D**) Densitometric analysis of Bcl-2 bands of sham, vehicle-treated HI and xyloketal B treated HI brains. Xyloketal B pretreatment increased the expression of Bcl-2 proteins 24 h after HI in comparison to the vehicle-treated HI group; (**E**) Densitometric analysis of Bax bands of sham, vehicle-treated HI- and xyloketal B-treated HI brains. Xyloketal B pretreatment decreased the expression of Bax protein 24 h after HI in comparison to the vehicle-treated HI group; (**F**) Densitometric analysis of the ratio of Bcl-2-to-Bax bands of sham, vehicle-treated HI and xyloketal B treated HI brains. Xyloketal B pretreatment increased the ratio of Bcl-2-to-Bax in comparison to the vehicle-treated HI group. *: *p* < 0.05, *n* = 3. Sham: Sham-surgery group; HI: Hypoxic-ischemic injury group; Xyl B + HI: hypoxic-ischemic injury plus pretreatment with xyloketal B group.

Caspase 3 and Bcl-2-to-Bax ratio are the major indicators of apoptosis. To understand whether neuroprotective effects of xyloketal B pre-treatment are mediated through suppression of apoptosis, we compared the levels of these apoptosis-associated proteins from ipsilateral brains after HI insult among sham, vehicle-treated HI and xyloketal B-treated HI groups. As shown in Figure 6A, cleaved caspase-3 (cleaved casp-3) protein expression significantly increased 24 h after HI (0.23 ± 0.01 arbitrary unit (AU), *n* = 3. *p* < 0.05) in comparison to that of the sham group (0.06 ± 0.01 AU, *n* = 3). Xyloketal B pretreatment significantly reduced the cleaved caspase-3 protein level after HI (0.15 ± 0.01 AU, *n* = 3) in comparison to the vehicle-treated HI group (0.23 ± 0.01 AU. *p* < 0.05) (Figure 6B).

Similarly, xyloketal B pre-treatment significantly increased Bcl-2 protein level (1.23 ± 0.06 AU, *n* = 3. *p* < 0.05) in comparison to the vehicle treated HI group (0.93 ± 0.08 AU, *n* = 3) (Figure 6C,D). On the other hand, Bax protein level in the vehicle-treated HI group (0.55 ± 0.02 AU, *n* = 3) was significantly greater than that of the sham group (0.27 ± 0.01 AU, *n* = 3), while xyloketal B pre-treatment significantly reduced Bax protein level (0.34 ± 0.02 AU, *n* = 3. *p* < 0.05) when compared to that of the vehicle-treated HI group (Figure 6C,E). The ratio of Bcl-2 to Bax protein significantly decreased in the vehicle treated HI group (1.62 ± 0.19 AU, *p* < 0.05) when compared to the sham group (3.34 ± 0.20 AU), while the ratio of Bcl-2 to Bax protein returned to the control level in the xyloketal B-treated group (3.47 ± 0.12 AU. *p* < 0.05) (Figure 6F). These results suggest that the apoptotic pathway is involved in HI brain injury. Xyloketal B pre-treatment decreased caspase-3 and Bax levels, and increased Bcl-2 levels and Bcl-2-to-Bax ratio, thus reducing HI-induced brain injury. The neuroprotective effect of xyloketal B may have been through the inhibition of pro-apoptotic signaling in HI brain injury.

### 2.5. Discussion

In this study, we reported that the novel marine compound Xyloketal B (1) reduced OGD-induced cell death in primary cortical cell culture *in vitro*; (2) reduced brain infarct volume and improved neurological functional recovery after hypoxic-ischemic brain injury in neonatal mice *in vivo*; (3) suppressed HI-induced apoptosis, and inhibited the expression of cleaved caspase-3 and Bax proteins, and increased the expression of Bcl-2 protein in HI brains; and (4) inhibited calcium entrance in cultured cortical neurons *in vitro*. Our findings collectively suggest that xyloketal B protects against neonatal stroke from hypoxic-ischemic brain injury, both *in vivo* and *in vitro*, and the neuroprotective effects of xyloketal B are largely mediated by the inhibition of apoptotic signaling and calcium entry during hypoxic-ischemic injury.

Xyloketal B is a newly discovered novel compound from mangrove fungus Xylaria sp [5,18]. This is the first study to demonstrate that xyloketal B exhibits neuroprotective effects in the neonatal HI brain injury mouse model *in vivo* and in the OGD model in primary cortical cell culture. The OGD protocol mimics cerebral ischemia-reperfusion injury *in vitro*. Xyloketal B in the range of 12.5–200 μM reduced OGD-induced cell injury in PC12 cell 24 h later [13]. In this study, cultured cortical neurons were first subjected to a short period of OGD followed by a prolonged period of re-oxygenation and return to normal culture medium. We found 100 μM xyloketal B significantly decreased propidium iodide (PI) fluorescence density in comparison to other control groups. Consistent with the previous study [13], our results showed that xyloketal B protected cortical neurons against OGD-induced cell death in culture.

The neonatal stroke mouse model [19] was modified from the Levine-Rice model [20,21] and hypoxic insult was applied following unilateral carotid ligation. This mouse model is commonly used for studying the neonatal HIE [20,21]. By 7 days of age, the brains of the mouse pups are histologically similar to that of 32- to 34-week gestation human fetus or newborn infant [22,23]. We showed for the first time that xyloketal B reduced both infarct volume (Figure 3) and promoted behavioral recovery (Figure 4) of mice following HI insult *in vivo*. Mitochondrial damage, free radical production, glutamate excitatory toxicity, calcium imbalance, oxidative stress, inflammation, caspase-3 activation, and apoptosis are involved in HIE. Neuronal cell death following hypoxic- ischemic insult is generally attributed to rapid excitotoxicity-induced necrosis and delayed apoptosis [24,25]. Accumulating data suggests that apoptotic mechanisms play a prominent role in ischemic brain injury in neonatal models [14,15] in comparison to adult models in rodents [26,27].

Apoptosis can be activated by two main pathways: an intrinsic pathway and an extrinsic pathway [28,29]. In this study, we tested the apoptotic mechanisms involved in hypoxic-ischemic injury in the neonatal brain [28,29]. Apoptosis involves the mitochondrial release of cytochrome C and apoptosis-inducing factor, which activates caspase-dependent and independent cell death pathways [30], respectively. Caspases are a unique family of cysteinyl-aspartate proteases that play an important role in the initiation and execution of apoptosis. Caspases are classified into three groups: group I contains caspase-1, -4, and -5; group II contains caspase-2, 3, and 7; and group III contains caspase-6, -8, and -9, and -10. Caspase-3 is the major executioner caspase in neurons [31]. Activation of caspase-3 results in proteolysis of essential cellular proteins, including cytoskeletal proteins and kinases, and leads the morphological changes including nuclear fragmentation. Caspase-3 protein level increased significantly after HI insults [24,32,33], and inhibiting caspase-3 activation provided neuroprotection in various rodent models of neonatal brain injury [26,34,35]. Consistent with previous reports, in this study, we showed that hypoxic–ischemic brain injury increased the number of apoptotic TUNEL-positive cells, and the level of caspase-3 protein. Xyloketal B significantly reduced the number of TUNEL-positive cells and the level of caspase-3 protein, suggesting that Xyloketal B prevented HI-induced apoptotic processes.

The mitochondria-dependent pathway leads to the initiation of apoptosis. The Bcl-2 protein family is a principal regulator of mitochondrial membrane integrity and function. It is classified into two major subgroups, anti-apoptotic proteins such as Bcl-2, and pro-apoptotic proteins such as Bax [36]. HI stress induces the accumulation and conformational changes of Bax, which leads to the opening of mitochondrial transition pores and release of cytochrome C. In the ischemic region, the level of Bcl-2 decreases and the level of Bax increases [37], leading to a release of cytochrome C from mitochondria. Cytochrome C initiates cell death following brain ischemia [18,31,38,39,40], and reducing Bax levels and/or increasing Bcl-2 levels suppresses the release of cytochrome C [41,42]. Once released into the cytosol, cytochrome C binds to Apaf-1 (apoptosis protease activating factor-1) and procaspase-9 to form the apoptosome, thereby activating caspase-9 and caspase-3, and initiating apoptotic cell death [41,42,43]. In this study, we also observed that the Bcl-2/Bax ratio was reduced after the HI insult, and xyloketal B pretreatment restored the balance of Bcl-2/Bax ratio. These findings support the notion that xyloketal B reduces mitochondria-dependent apoptosis by preventing the reduction of the Bcl-2-to-Bax ratio, and in turn the activation of caspase-3.

A cytotoxic accumulation of intracellular calcium ions is a key factor for anoxic or ischemic neuronal death [16,17,44]. It initiates a series of cytoplasmic and nuclear events, including triggering the intrinsic apoptotic pathway through the activation of caspases [45]. We found that xyloketal B reduced calcium entry, suggesting that it has multiple cellular targets that contribute to its overall neuroprotective effects. Ischemic-hypoxic insult causes neuronal excitotoxicity and leads to enhanced calcium level through multiple voltage-gated and ligand-gated calcium conducting channels [4]. Further studies need to be carried out to identify the calcium-conducting channels that are sensitive to xyloketal B.

Functional behavioral recovery from cerebral ischemic insults is considered as one of the key standards in identification of potential therapeutics. In this study, we adapted body weight and a number of behavioral tests, including evaluation of geotaxic reflex [46], cliff avoidance reaction [47], and grip test [48], and reported that xyloketal B improved sensorimotor functional recovery after HI. Therefore, xyloketal B has opened the possibility of using marine drugs as pharmacological interventions of the stroke field.

## 3. Experimental Section

### 3.1. Animals

Timed-pregnant CD1 mice were purchased from Charles River Laboratories (Sherbrooke, QC, Canada). Pups were considered the postnatal day 0 (P0) on the day of birth. All experiments using these animals strictly followed the guidelines of the Canadian Council on Animal Care (CCAC protocol) in science and all animal experimental procedures were approved by the local Animal Care and Use Program Committee, Office of Research Ethics at the University of Toronto.

### 3.2. Reagents and Drugs

Dimethyl sulfoxide (DMSO), 2,3,5-triphenyltetrazolium chloride (TTC), cresyl violet, and propidium iodide (PI) were purchased from Sigma-Aldrich (Sigma-Aldrich, St. Louis, MO, USA) and Hank’s Balanced Salt Solution (HBSS), and trypsin was purchased from Gibco (Gibco, Life Technologies, Burlington, ON, Canada). Xyloketal B was a gift from Department of Pharmacology, Zhongshan School of Medicine, Sun Yat-Sen University, China.

### 3.3. Primary Cell Culture and Oxygen-Glucose Deprivation (OGD) of Cultured Cortical Neurons

Embryonic cortical cultures were prepared from E16 CD1 mice [49]. Specifically, cortical tissue was digested with 0.025% Trypsin/EDTA at 37 °C for 15 min and washed with Neurobasal medium supplemented with 10% FBS, 1.8% HEPES, 0.25% Glutamax, and 1% antibiotic-antimyocotic. The cortical cell suspension was triturated, filtered, and then centrifuged (1000 rpm, 5 min). Cell pellets were then resuspended in the Neurobasal culture medium and plated at the desired density of 100,000 cells/well in 100 μL of medium. All cultures were kept at 37 °C in a humidified 5% CO_2_-containing atmosphere.

Cultured cortical cells were used for OGD experiments on the 7th days *in vitro* (DIV7) [44,50]. Cells were pretreated with various concentrations of xyloketal B (10, 30, or 100 μM in DMSO, 0.1% vol/vol) or DMSO only (vehicle, 0.1% vol/vol) for the designated time. The culture medium was then replaced by either deoxygenated glucose-free bicarbonate solution, containing (mM) 121 NaCl, 5 KCl, 1 Na-pyruvate, 1.8 CaCl_2_, 25 NaHCO_3_, and 0.01 glycine (pH to 7.4 with HCl; deoxygenated by bubbling N_2_) or the control solution containing (mM) 121 NaCl, 5 KCl, 20 D-glucose, 10 HEPES-acid, 7 HEPES-Na salt, 3 NaHCO_3_, 1 Na-pyruvate, 1.8 CaCl_2_, and 0.01 glycine (pH 7.4 with NaOH) for 30 min. The cultures were maintained in an anaerobic chamber containing a 5% CO_2_, 10% H_2_, and 85% N_2_ (0.2% O_2_) atmosphere for 90 min at 37 °C. The plates were returned to the normoxic incubating conditions for 24 h after the normal culture medium containing either xyloketal B or vehicle.

Propidium iodide staining. The cultured cells were incubated with propidium iodide (PI, 1 μg/mL final concentration) at room temperature for 20 min. The fluorescence density of PI was measured using a multiwall plate fluorescence scanner (Synergy H1 Microplate Reader, Biotek, Winooski, VT, USA) with excitation/emission wavelengths at 488/630 nm as described previously [44,50].

### 3.4. Fura-2 Calcium Imaging

Intracellular calcium ([Ca]_i_) was measured using a Fura-2 ratiometric Ca^2+^ imaging system as described previously [51,52]. Neurons were pre-loaded with Fura-2 AM (2μ M) in the dark for 40 min at room temperature. Fura-2 calcium signal was acquired at alternate excitation wavelengths of 340 and 380 nm by a Deltaram V single monochromator (PTI, Edison, NJ, USA) controlled by EasyRatioPro (PTI, Edison, NJ, USA) and digitized by an intensified charged-coupled device (ICCD) camera (PTI, Edison, NJ, USA) and fluorescence intensity (Poenie-Tsien) ratios of images of 340/380 nm were calculated with EasyRatioPro (PTI, Edison, NJ, USA). The composition of solutions was as follows (mM): 140 NaCl, 2 CaCl_2_, 1 MgCl_2_, 10 HEPES, 10 glucose, and 4 (basal) or 20 (high potassium) KCl with or without 100 μM xyloketal B with pH of 7.3–7.4 and osmolality ranging from 320 to 330 mOsm.

### 3.5. Drug Administration

Animals were randomly assigned to different treatment groups for the *in vivo* experiment and the experimenters were blinded to treatment group during all the experimental procedures. Thirty minutes prior to the onset of HI, xyloketal B was dissolved in 1 × PBS with 2% dimethyl sulfoxide (DMSO) and administrated intraperitoneally at a dose of 5 mg/kg body weight in 0.1 mL (treatment group). For vehicle controls, and equal volume of 2% DMSO (vol/vol) was administered in the same way. The experimental protocol is shown in Figure 2.

### 3.6. Hypoxic-Ischemic Brain Injury Model

The Rice-Vannucci neonatal adaptation of Levine [20,21] procedure with some modifications was used to induce hypoxic-ischemic brain injury in neonatal mice [19]. Specifically, 7 days-old mouse pups were anesthetized with 3% isoflurane in a balance of oxygen, as described previously [19]. All surgical procedures were carried out using a stereo dissecting microscope (SMZ-2B Nikon, Tokyo, Japan). A 0.5-cm midline cervical incision was made in the anterior neck. The right common carotid artery was occluded by bipolar electrical coagulation (Vetroson V-10 Bi-polar electrosurgical unit, Summit Hill Laboratories, Tinton Falls, NJ, USA), and the skin incision was closed with tissue glue (3M Vetbond, 3M Animal Care Products. St. Paul, MN, USA). Body temperature was monitored and controlled using a homeothermic blanket control unit (K-017484 Harvard Apparatus, MA, USA) during the procedure. The pups were placed in a clean cage under a heating lamp until fully awake, and then returned to their dam.

For the hypoxic insult, the pups were relocated to a hypoxic chamber (A-Chamber A-15274 with ProOx 110 Oxygen Controller/E-720 Sensor, Biospherix, NY, USA) perfused with a humidified gas mixture containing 8% oxygen balanced with 92% nitrogen with a constant flow rate of 100 mL/min. Oxygen concentration was regulated by a compact oxygen controller (ProOx 110 controller, Biospherix, NY, USA), to which a compressed nitrogen gas source (Linde, Mississauga, ON, Canada) was attached. Body temperature was again monitored and maintained. After hypoxia, mouse pups were recovered on a heating pad (33 °C) for 30 min and then returned to their mother for recovery.

For sham animals, a median neck incision was performed and the right common carotid artery was exposed, but the common carotid artery was not occluded and nor exposed to hypoxia.

### 3.7. Functional Recovery Assessments

Neurological damage imposed by hypoxic-ischemic brain injury can lead to sensorimotor impairments. Body weight and sensorimotor performance (geotaxis reflex, cliff aversion reaction and grip test) performance were examined 1 day, 3 days and 7 days after the HI procedure by two researchers in a blinded manner. All animals performed the tasks in the above cited order. All trials were video recorded and scored by two different researchers blinded to experimental group.

(1) Geotaxis Reflex is an automatic, stimulus-bound orientation movement considered diagnostic of vestibular and/or proprioceptive function [46]. At 1 day, 3 days and 7 days after the HI procedure, animals were placed head down in the middle of an inclined 30 cm board (angle of 30°). The latency to make a 90° turn was recorded up to a maximum time of 60 s and each mouse was tested 2 times.

(2) Cliff Aversion Reaction is used to assess maladaptive impulsive rodent behavior [47]. The apparatus consisted of a wood board (50 × 25 × 2.5 cm, L × W × H), with one end protruding 15 cm over the edge of a desk. At 1 day, 3 days and 7 days after the HI procedure, animals were gently placed on the protruding end of the board with their head down and forepaws off the board, and the latency to place both their forepaws back on the board was measured up to a maximum time of 60 s and each mouse was tested 2 times.

(3) Grip Test Forepaw grip test was performed to assess force and fatigability [48]. At 1 day, 3 days and 7 days after the HI procedure, animals were suspended by both forepaws on a metallic rope (diameter, 1.5 mm) stretched horizontally 50 cm over a cotton pad. Time before falling was recorded (maximum: 60 s) and each mouse was tested 2 times.

### 3.8. General Histology and Infarct Size Measurement

TTC straining. Brains were sectioned coronally into ~1 mm slices and immersed in 1% TTC at 37 °C in the dark for 20–30 min, 24 h after HI procedure. The areas of ipsilateral and contralateral hemispheres were measured using Image J software (National institute of Health, Bethesda, MD, USA). After correcting for edema, the volumes of infarction were calculated as follows: Corrected infarct volume (%) = [contralateral hemisphere volume − (ipsilateral hemisphere volume-infarct volume)]/contralateral hemisphere volume × 100% as described in the study [19].

Nissl staining. Brain slices (30 μM) used for Nissl staining (1% cresyl violet, 2 min) were made 7 days after the HI procedure.

### 3.9. Terminal Deoxynucleotidyl Transferase dUTP Nick-End Labeling (TUNEL) Assay

Mouse brains, collected 3 days after HI, were fixed with 4% formaldehyde, embedded in agarose, and sectioned at a thickness of 30 μM on a Series 1000 sectioning system (The Vibratome Company, St. Louis, MO, USA). In order to quantify the number of apoptotic cells, two coronal brain sections from each brain were used. Apoptotic cells were detected by TUNEL assay using an ApopTag^®^ Red *In situ* Apoptosis Detection Kit (S7165, Chemicon International Inc., Temecula, CA, USA) according to the manufacturer’s instructions. After the TUNEL staining, the slices were mounted using ProLong Gold antifade reagent (Invitrogen, Carlsbad, CA, USA). The brain slices were then imaged with a Zeiss Examiner D1 microscope in conjunction with Zeiss microscope software (Carl Zeiss Microscopy GmbH, Jena, Germany), and the number of apoptotic cells was counted per ×10 field.

### 3.10. Western Blots Analysis

Protein extraction and Western Blot were conducted as described previously [49,50]. Proteins extracted from brain tissues collected 24 h after HI were used for Western blot analysis. Specifically, protein extracts (30 μg) were separated on 12% SDS-PAGE gels that were subsequently transferred to polyvinylidene difluoride (PVDF) membrane (400 mA, 1.5 h). Blocking of membranes (5% non-fat dry milk), washes (Tris buffered saline, TBS) and secondary antibody incubations were all performed at room temperature, whereas primary antibodies were incubated overnight at 4 °C (anti-cleaved caspase-3, 1:500; Bcl-2, 1:1000; Bax, 1:500; Cell Signaling Technology, Danvers, Mass. β-actin, 1:10,000; GAPDH, 1:10,000, Sigma-Aldrich, St. Louis, MO, USA). Signals were developed using enhanced chemiluminescent reagents (PerkinElmer, Waltham, MA, USA) and analyzed by exposure to film (HyBlot CL, Denville Scientific Inc., South Plainfield, NJ, USA).

### 3.11. Statistical Analysis

Statistical analyses were performed using the Statistical Package for the Social Sciences (SPSS, Chicago, IL, USA) 13.0 software. Quantitative data were expressed as mean ± standard error of the mean (SEM). Multiple group analysis was performed using one-way analysis of variance (ANOVA) followed by Fisher-LSD (least significant difference) post hoc or Dunnett’s T3 for multiple pairwise comparisons. *P* values of less than 0.05 (*p* < 0.05) were considered statistically significant. All experiments and analysis were performed in a blinded manner, as the experimenters were not aware of the treatment conditions.

## 4. Conclusions

Hypoxic-ischemic injury to the central nervous system can have devastating lifelong effects on the developing fetus and the neonate. To the best of our knowledge, this study is the first demonstration of the neuroprotective effect of xyloketal B on neonatal hypoxic-ischemic brain injury both *in vivo* and *in vitro*. Given that it is a natural marine compound and shows minimal toxicity in a variety of cell systems in previous studies, xyloketal B has potential as a therapeutic for the treatment or prevention of neonatal hypoxic-ischemic brain injury and its related brain disorders, such as hypoxic-ischemic encephalopathy.

## Abbreviations

DAPI: 4′,6-diamidino-2-phenylindole
DIV: day *in vitro*
DMSO: dimethyl sulfoxide
MPP+: 1-methyl-4-phenylpyridinium
OGD: oxygen-glucose deprivation
PI: propidium iodide
PVDF: polyvinylidene difluoride
